# Mycorrhizal helper bacteria further promote mycorrhizal fungi to improve cold tolerance in rice seedlings: evidence from oxidative stress, osmoregulation, photosynthesis, and related genes in rice

**DOI:** 10.3389/fpls.2025.1692304

**Published:** 2025-10-15

**Authors:** Feng Shi, Fangyuan Liu, Xue He, Siyu Zhu, Honghe Li, Yiwen Ding, Bo Zhang, Tianle Xu, Fuqiang Song

**Affiliations:** ^1^ Engineering Research Center of Agricultural Microbiology Technology, Ministry of Education & Heilongjiang Provincial Key Laboratory of Ecological Restoration and Resource Utilization for Cold Region & Key Laboratory of Microbiology, College of Heilongjiang Province & School of Life Sciences, Heilongjiang University, Harbin, China; ^2^ Heilongjiang Academy of Agricultural Sciences, Harbin, China; ^3^ Heilongjiang Vocational and Technical College of Architecture, Harbin, China

**Keywords:** AMF, rice, cold stress, physiological response, mycorrhizal helper bacteria

## Abstract

**Introduction:**

Cold stress critically threatens rice productivity, necessitating innovative strategies to enhance seedling resilience. While arbuscular mycorrhizal fungi (AMF) and associated bacteria synergistically improve plant stress tolerance, their collaborative mechanisms in rice cold adaptation remain underexplored.

**Methods:**

Here, we developed a composite inoculant combining *Rhizophagus intraradices* (Ri) with *Agrobacterium rhizogenes* (Ar) and *Bacillus subtilis* (Bs) to investigate their synergistic effects under graded cold stress (25–4 °C).

**Results:**

The Ri+Ar+Bs (RAB) consortium elevated mycorrhizal colonization by 17% (reaching 87.5%) and synergistically promoted plant growth, increasing height and root length by 9.56% and 43.7%, respectively, under 4 °C stress compared to Ri alone. RAB enhanced antioxidant capacity (24.9% higher SOD activity, 12.37-fold CAT activity) and proline accumulation (78.4%), reducing malondialdehyde (43.7%) and electrolyte leakage (13.64%). Hormonal equilibrium was maintained via upregulated indole-3-acetic acid and gibberellic acid levels. Photosynthetic performance improved significantly (11.29% higher net rate at 4 °C), supported by activation of *OsHBP1b* and *CBF1*. Concurrently, RAB upregulated cold-tolerance genes (*LTG5RT, OsDREB1A*), with functional specialization observed.

**Discussion:**

Ar amplified Ri-mediated height improvement and gene expression, while Bs enhanced root development and photosynthetic efficiency. These findings advance microbial consortia design for climate-resilient agriculture, offering actionable strategies to safeguard rice productivity under extreme cold.

## Graphical Abstract

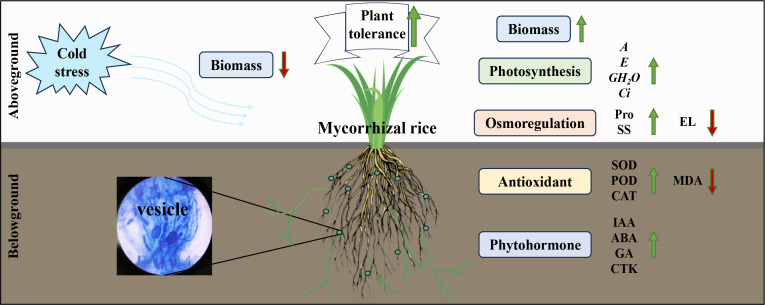

## 1 Introduction

Rice (*Oryza sativa* L.) is a staple crop for more than half of the global population, and its stable and high yield is critical for ensuring food security (Savary et al., 2020). However, rice seedlings are highly sensitive to low temperatures, and cold stress can compromise cell membrane integrity, inhibit photosynthesis, and disrupt metabolic balance (Feng et al., 2023). This leads to growth stagnation and even plant mortality, ultimately resulting in a significant reduction in tiller number, spikelet number, and final yield (Islam et al., 2023). The mechanisms by which cold stress induces plant damage primarily include the following aspects: First, cold stress disrupts the liquid-crystalline structure of the cell membrane, affecting its integrity and functionality (Pradhan et al., 2019). This leads to the leakage of ions and cytoplasm, thereby compromising cellular stability (Ding et al., 2019). Secondly, cold stress induces cell dehydration and water imbalance within plant tissues, resulting in a decrease in turgor pressure and triggering processes such as cell apoptosis (Gusain et al., 2023). Cold stress disrupts the redox balance within plants, leading to the excessive production and accumulation of reactive oxygen species (ROS), which subsequently triggers oxidative stress responses and damages the cell membrane (Hassan et al., 2021). Finally, cold stress interferes with the physiological and metabolic processes of plants, impacting critical pathways such as energy metabolism and photosynthesis (Engelberth et al., 2019). Consequently, developing reliable strategies to improve cold tolerance in rice seedlings represents an urgent research priority.

Arbuscular mycorrhizal fungi (AMF), forming symbiotic associations with approximately 80% of terrestrial plants, are among the most ubiquitous and agriculturally significant microorganisms in soil ecosystems (Zhang et al., 2023a). For instance, *Rhizophagus intraradices* has been demonstrated to enhance ROS scavenging, mitigate membrane lipid peroxidation, and improve membrane stability by augmenting root water uptake capacity (Liu et al., 2014). It further induces the upregulation of antioxidant enzymes, including superoxide dismutase (SOD) (Pasbani et al., 2020), stimulates the accumulation of osmolytes such as soluble sugars and proline (Zhu et al., 2015), and decelerates chlorophyll degradation to sustain photosynthetic efficiency under stress conditions (Wei et al., 2023). AMF can regulate the expression of host plant-related genes through symbiotic relationships to complete various physiological and biochemical processes. Studies have shown that AMF can regulate related metabolic pathways by overexpressing genes such as *CsPT1–11* in cucumber seedlings, increasing antioxidant enzyme activity, and enhancing the cold tolerance of cucumber seedlings (Ma et al., 2018). AMF can also affect the metabolism and antioxidant-related metabolites (such as flavonoids and lipids) in *Elymus nutans* to regulate the cold tolerance of gramineous plants (Zhang et al., 2023b). It can be seen that AMF can enhance the plant’s adaptability to low-temperature environments by improving the plant root structure and physiological functions, thereby improving the plant’s cold tolerance. Therefore, it has great application potential in improving the cold stress tolerance of rice. Nevertheless, the potential synergistic effects of AMF and mycorrhizal helper bacteria (MHB) on enhancing cold tolerance in host plants remain insufficiently explored, warranting further investigation.

MHB facilitate the colonization of host plants by mycorrhizal fungi, thereby enhancing the establishment and functionality of mycorrhizal symbioses (Yang et al., 2023b). Among, them *Agrobacterium rhizogenes* (Ar) and *Bacillus subtilis* (Bs) have been widely studied and applied as MHB. *Agrobacterium rhizogenes* is a soil bacterium with a very wide range of infestation, capable of infesting a large number of plants and inducing them to differentiate a large number of hairy roots, which promotes the uptake of nutrients and enhances the resistance of the plant (Hashem et al., 2016). It has been shown that *Agrobacterium* can act as a mycorrhizal helper bacterium to directly promote AMF spore germination, mycelial growth, and AMF colonization in plant roots (Frey-Klett et al., 2007). *Bacillus subtilis* is widely distributed in the soil, has a powerful ability to survive, growth, reproduction speed, and nutritional conditions do not require high, can inhibit a variety of diseases, and at the same time can secrete a large number of metabolites conducive to plant growth (Huang et al., 2023). Studies have shown that Bs can be used jointly with AMF to promote plant growth, especially significantly increasing the biomass of wheat (Yadav et al., 2021). In our previous research, we found that combining AMF with mycorrhizal helper bacteria such as *Agrobacterium rhizogene*s can increase the AMF colonization rate of rice seedlings under salt stress and enhance the antioxidant enzyme activity and osmotic adjustment substance content of rice seedlings. It has ability to tolerate salt stress (Zhang et al., 2023a). However, it remains to be studied whether mycorrhizal helper bacteria can further promote AMF and improve the cold tolerance of host plants.

This study focuses on rice as the model organism and investigates the effects of different microbial strain co-inoculations on rice seedlings under simulated cold stress conditions. It also explores how MHB can further enhance the functionality of AMF under these conditions. Based on this, we hypothesize that co-inoculation with microbial strains can enhance the cold tolerance of rice seedlings under cold stress conditions by increasing their biomass, antioxidant enzyme activity, and osmotic regulation compound content, while also boosting photosynthetic efficiency and overexpressing cold tolerance-related genes in rice. These results provide insights into the enhanced cold tolerance of rice seedlings through the synergistic effects of two mycorrhiza helper bacteria, further promoting the role of Ri in improving cold resistance. The findings offer a theoretical basis for the development of Ri-based composite microbial inoculants and their application in rice seedling cultivation in high-latitude regions.

## 2 Materials and methods

### 2.1 Experimental materials

Rice (Oryza sativa L.) variety used in this experiment was selected as Suijing 18, a cold-intolerant variety mainly cultivated in northeastern China. Rice seeds were purchased from the Heilongjiang Provincial Academy of Agricultural Sciences.

Strain varieties used in this experiment were selected as: (1) *Rhizophagus intraradices* (Ri, isolate number: CGMCC No.10607): (obtained by pot expansion using sorghum as host, spore density is approximately 67/g); (2) *Bacillus subtilis* (Bs): (bacteria liquid, concentration is approximately 3×10^5^ CFU/mL); (3) *Agrobacterium rhizogenes* (Ar): (concentration is approximately, 3.0×10^5^ CFU/mL). The above strains were preserved by the Ecological Restoration Research Laboratory of Heilongjiang University.

The inoculants were inoculated as follows: the Ri inoculants (5%, w/w) were mixed with the rice seedling soil and used as the rice seedling substrate; the germinated rice seeds were soaked in the Ar bacteria liquid with a rice seed to bacterial liquid ratio of 1:1 (w/v), and a soaking time is 8–16 hours; the soaked seeds were sown in the seedling substrate; and after diluting 100 mL of Bs bacteria liquid into 500 mL of bacteria liquid with sterile water, the solution was sprayed evenly on the surface of the seeds (one seedling tray needed 500 mL of Bs bacteria liquid, i.e., 15 L/hm^2^). Thus, the inoculation of the AMF compound inoculants was completed.

### 2.2 Experimental design

The experiment was conducted in the light culture room of the School of Life Sciences of Heilongjiang University in May 2023. Rice seeds with full grains were selected and disinfected with NaClO with a concentration of 5% for 10 minutes and then washed 5 times with sterile water. The seeds were put into a constant temperature incubator at 30 °C for germination. When the germination rate reaches more than 80%, the strain inoculation process was completed. Then the seeds were cultured in a seedling pot (upper diameter: 12 cm: lower diameter: 10 cm, height: 13 cm).

There were two influencing factors: bacterial agent addition and low-temperature stress. Because it was found in previous experiments that adding two mycorrhizal helper bacteria, Ar and Bs, alone had no significant effect on the cold tolerance of rice seedlings. Therefore, in this study, the bacterial agent addition treatments were: Ri (only Ri bacterial agent is added), RA (RA, Ri, and Ar bacterial agents are added), RB (RB, Ri bacterial agent is added), and RAB (RAB, adding Ri, Ar, and Bs inoculants). Simultaneously, equal amounts of sterilized substrates of each microorganism were added to all groups to ensure that the observed differences were solely attributed to the microorganisms rather than the nutrient composition of the culture medium. Cold stress on this experiment were determined as four levels of treatments: 4 °C, 8 °C, 12 °C, and 25 °C.

There were a total of 16 treatment combinations, with 5 replicates for each treatment, arranged in random blocks, with 5 seedlings per pot, a total of 100 pots, and each pot was filled with 0.75 kg of matrix soil. They were placed into four low-temperature light incubators, respectively. The culture conditions are 12 hours light (25 °C) and 12 hours dark (25 °C) cycle, and the humidity is 80%. When the seedlings have grown for 30 days, the incubator was adjusted to 4 °C, 8 °C, 12 °C, and 25 °C within 24 hours. After reaching the target temperature, they were incubated for 24 hours and then adjusted the incubator to return to the temperature within 24 hours. At 25 °C, the stressed rice seedlings were restored and cultured for 7 days (Liu et al., 2014), and the rice seedlings were cultured for a total of 40 days. Then rice seedlings were sampled to detect various parameters.

### 2.3 Experimental methods

#### 2.3.1 Measurement of rice mycorrhizal colonization rate

On the 40th day after transplanting, the rice seedlings was in the rapid growth period. Rice seedlings are most sensitive to cold stress during this period and can easily reflect the cold tolerance of rice throughout the seedling stage. Rice seedlings showed signs of yellowing and dying under cold stress at 4 °C, which was representative, so samples collected samples at this time for measurement before the stress.

Fifty to one hundred root segments in each treatment were randomly selected, stained with the Trypan blue staining method. These segments were transparented, stained, decolorized, sliced, and observed under a 10×40 microscope. The mycorrhizal colonization rate was calculated as follows (Sun et al., 2022).


The colonization(%)=(number of mycorrhizal segments-positive segments)/(total number of segments)×100%

#### 2.3.2 Measurement of rice biomass

Five samples of rice were taken from each treatment after recovery culture, separated the above-ground part and roots of the plants, cleaned the roots with distilled water, and then washed them 3 times with deionized water. And the filter papers were used to absorb the surface moisture. Finally, the plant height and root length were also measured.

#### 2.3.3 Measurement of rice antioxidant enzyme and malondialdehyde content

The superoxide dismutase kit (Solarbio LIFE SCIENCES, BC0175), peroxidase kit (Solarbio LIFE SCIENCES, BC0095), catalase enzyme kit (Solarbio LIFE SCIENCES, BC0205), and malondialdehyde kit (Solarbio LIFE SCIENCES, BC0025) were used for measurement of rice leaf-related enzyme activities according to manufacturer’s instructions.

#### 2.3.4 Measurement of rice osmotic adjustment substance content and EL

The proline kit (Solarbio LIFE SCIENCES, BC0295) and soluble sugar kit (Solarbio LIFE SCIENCES, BC0035) were used to measure the enzyme activities related to rice leaves according to the manufacturer’s instructions. The electrolyte leakage rate of rice leaves was measured using the conductivity measurement method (Elkelish et al., 2020).

#### 2.3.5 Measurement of phytohormone content in rice

According to the manufacturer’s instructions, the contents of the respective hormones in rice leaves were measured using the following kits: Plant Auxin Kit (Beijing Boao Tuoda Technology Co., Ltd., TOPEL03461, Beijing, China), Abscisic Acid Kit (Beijing Boao Tuoda Technology Co., Ltd., TOPEL03473, Beijing, China), Gibberellin Kit (Beijing Boao Tuoda Technology Co., Ltd., TOPEL03457, Beijing, China), and Cytokinin Kit (Beijing Boao Tuoda Technology Co., Ltd., TOPEL12026, Beijing, China). The principle of all the kits is based on the double-antibody, one-step sandwich enzyme-linked immunosorbent assay (ELISA). After obtaining the respective standard curves, rice leaves were homogenized with the corresponding extraction solution. The appropriate reagents were then added, and the analysis was performed using a microplate reader at 450 nm wavelength.

#### 2.3.6 Measurement of rice photosynthetic gas exchange parameters

Photosynthetic gas exchange parameters were measured using an LI-6400 photosynthesis instrument (LI-6400XT, LI-COR Corporate, Lincoln, Nebraska USA, USA). On June 12, 2023 (40 days of growth), from 9:00 to 11:00 a.m., plants with consistent growth were selected in each treatment to measure the relevant indices, and each treatment was measured three times. The net photosynthetic rate (*A*), stomatal conductance (*GH_2_O*), intercellular CO_2_ concentration (*Ci*), and transpiration rate (*E*) were measured.

#### 2.3.7 Rice leaf RNA extraction, cDNA synthesis, and quantitative reverse transcription

Fresh rice leaves were ground in liquid nitrogen, and RNA was extracted using the plant RNA extraction kit (FreeZol Reagent (Vazyme Biotech Co., Ltd)). Its integrity and purity were verified with the NanoDrop 2000c (Thermo Scientific, Pittsburgh, PA, United States) system. Reverse transcription was then performed using the reverse transcription kit (HiScript^®^ III RT SuperMix for qPCR (+gDNA wiper) (Vazyme Biotech Co., Ltd)).

The primer sequences of related genes were quoted from previous research results (**Supplementary Table S1**), and the primers were synthesized by Beijing Sangon Biotech. Configure the reaction system according to the template tracking dye method quantitative PCR detection kit (ChamQ^®^ Universal SYBR qPCR Master Mix (Vazyme Biotech Co., Ltd)). Each real-time fluorescence quantitative polymerase chain reaction mixture consists of 10 μL of 2×ChamQ SYBR qPCR Master Mix, 0.8 μL of primers, 1 μL of undiluted cDNA stock solution, and 8.2 μL of ddH_2_O. The total reaction system was 20 μL. The REFA40425 fluorescence quantitative PCR instrument (Kote Technology Holdings Co., Ltd.)was used to perform the qRT-PCR reaction. And the reaction conditions were selected according to the instructions (**Supplementary Table S2**) and the temperature required for the relevant primers. The relative expression of transcript samples was analyzed using the 2^−ΔΔct^ method, and each treatment was repeated three times.

### 2.4 Data processing and analysis

SPSS 25 was used for data processing and statistical analysis. The ggplot2 and ggbiplot2 software packages in Origin 2021 PRO and R software (4.3.2) were used for drawing. The data was tested to see if it conforms to the normal distribution. If not, log transformation was performed. One-way ANOVA and two-way ANOVA were used to test the significance of each treatment. The Tukey test was used at 0.05. The significance of differences between groups at different levels was tested. Structural equation modeling (SEM) and multivariate statistical methods were used to conduct hypothesis testing on complex path relationship networks. The *lavaan* package in R software was used to detect the direct and indirect effects of inoculant addition and temperature treatment on rice seedling growth indicators and physiological and biochemical indicators. *A priori* model based on a review of the literature and our knowledge of how these predictors are related was constructed.

## 3 Results

### 3.1 Effects of different fungal strain combinations on the mycorrhizal colonization rate of rice seedlings

**Figure 1:**
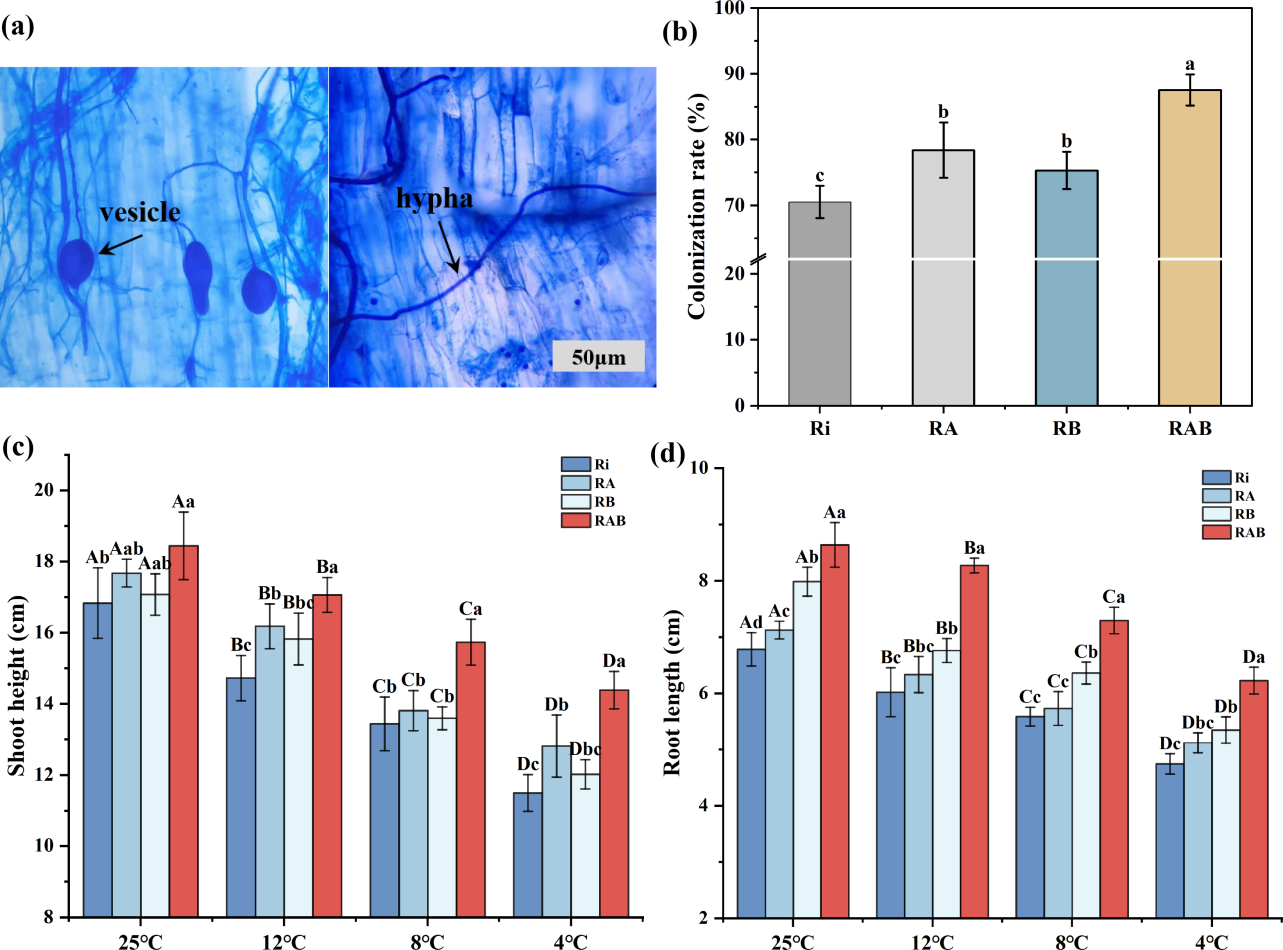
Ri colonization rate and colonization status in rice roots and rice biomass. **(A)** Vesicle and hyphal structures in the RAB treatment group compared to the Ri treatment group; **(B)** Colonization rate among different treatment groups; **(C)** Plant height of rice seedlings; **(D)** Root length of rice seedlings. Results are presented as mean ± standard deviation from five replicates. Different letters indicate significant differences between treatments (*P* < 0.05).

Analysis of mycorrhizal colonization characteristics (**Figure 1**) revealed that, after 40 days of cultivation, a stable symbiotic relationship had been established between Ri and the rice roots in the different strain combinations. Microscopic observation confirmed that the RAB treatment group formed typical AMF structures within the roots, including clearly visible vesicles and an extensive hyphal network. Quantitative analysis of mycorrhizal colonization rates indicated significant differences among the treatment groups (*P* < 0.05). The RAB treatment group exhibited a colonization rate of 87.5%, which was significantly higher than that of the Ri (+17%), RA (+9.1%), and RB (+12.2%) treatment groups (*P* < 0.05). These results demonstrate that the synergistic effect of the microbial strains can significantly enhance colonization efficiency.

Analysis of growth parameters following cold stress treatment indicated that, under normal temperature conditions, the RAB treatment group exhibited a significant increase in plant height, with values 9.56%, 4.34%, and 8.01% higher than those of the Ri, RA, and RB treatment groups, respectively (*P* < 0.05). In the cold stress gradient experiment, plant height exhibited a significant decreasing trend as the stress temperature lowered (*P* < 0.05). However, the RAB treatment group consistently maintained a significant advantage across all temperature conditions (*P* < 0.05). It is noteworthy that at both 12 °C and 4 °C, the plant height of the RA group was significantly higher than that of the Ri group (*P* < 0.05), whereas no significant difference in plant height was observed between the RB and Ri groups (*P*>0.05). This indicates that the RA combination more effectively promoted the increase in rice plant height compared to the RB combination.

Root development data indicate that, under all temperature conditions, the RAB treatment group exhibited significantly greater root length compared to the Ri, RA, and RB groups. Although cold stress significantly reduced root length (*P* < 0.05), it is noteworthy that at 12 °C, 8 °C, and 4 °C, the RB group showed significantly greater root length than the Ri group (*P* < 0.05). At the same temperatures, no significant difference in root length was observed between the RA and Ri groups (*P*>0.05), suggesting that the RB combination more effectively promoted the increase in rice root length than the RA combination.

### 3.2 Effects of different strain combinations on antioxidant enzyme activity and MDA content of rice under cold stress

Malondialdehyde (MDA) content was significantly positively correlated with the stress intensity (*P* < 0.05). As the temperature decreased from 25 °C to 4 °C, MDA content in the Ri group increased by 3.4 times, whereas the RAB treatment significantly inhibited MDA accumulation in rice (*P* < 0.05). At 4 °C, the MDA content in the RAB group was 43.7% lower than that in the Ri group, and it was significantly better than that of the other treatment groups (*P* < 0.05). These results suggest that the composite treatment possesses the best optimal peroxide scavenging ability.

**Figure 2:**
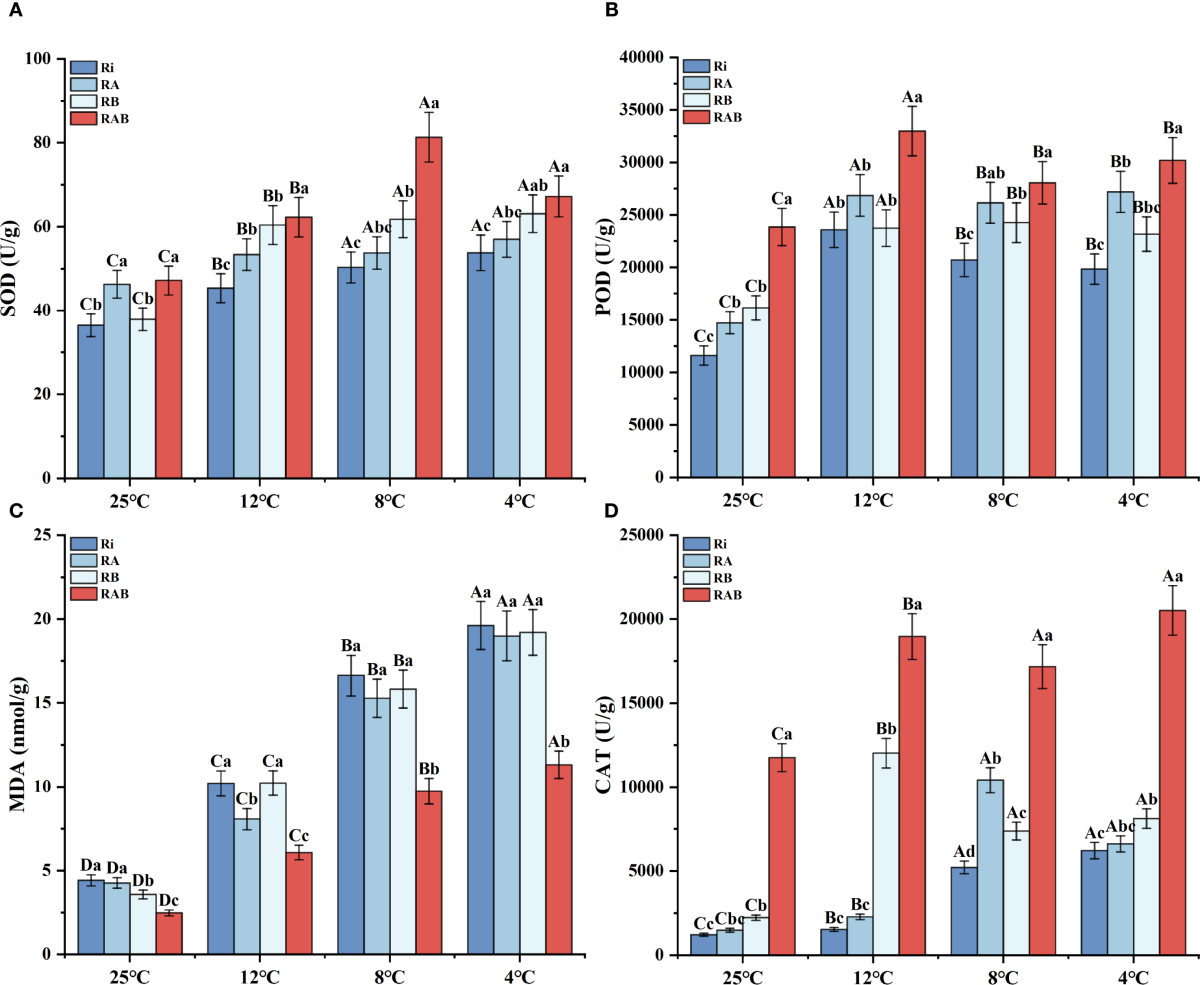
Antioxidant enzyme activity and MDA content in rice seedling leaves. Note: **(A)** SOD; **(B)** POD; **(C)** MDA; **(D)** CAT. Results are presented as mean ± standard deviation from five replicates. Uppercase letters indicate differences between temperatures (*P* < 0.05), while lowercase letters represent differences between different fungal treatments at the same temperature (*P* < 0.05).

Analysis of the antioxidant enzyme system response indicated that the activity of SOD increased linearly with the intensification of stress (**Figure 2A**). At 4 °C, the SOD activity in the RAB group reached its peak, showing a 24.9% increase compared to the Ri group (*P* < 0.05). Catalase (CAT) activity exhibited a dynamic pattern, initially increasing and then stabilizing (**Figure 2B**). At 12 °C, the CAT activity in the RAB group was 12.37 times that of the Ri group (*P* < 0.05), and even under extreme cold stress (4 °C), it maintained a 3.03-fold advantage. The variation in peroxidase (POD) activity exhibited temperature specificity. At 12 °C, all treatment groups reached peak activity, with the RAB group showing an 81.3% increase compared to the Ri group (*P* < 0.05). However, at 4 °C, activity decreased to 76.5% of the level observed at normal temperature. Furthermore, a comprehensive analysis revealed that the antioxidant capacity of the RA group was superior to that of the RB group.

### 3.3 Effects of different strain combinations on rice osmotic regulatory substances and EL under cold stress

**Figure 3:**
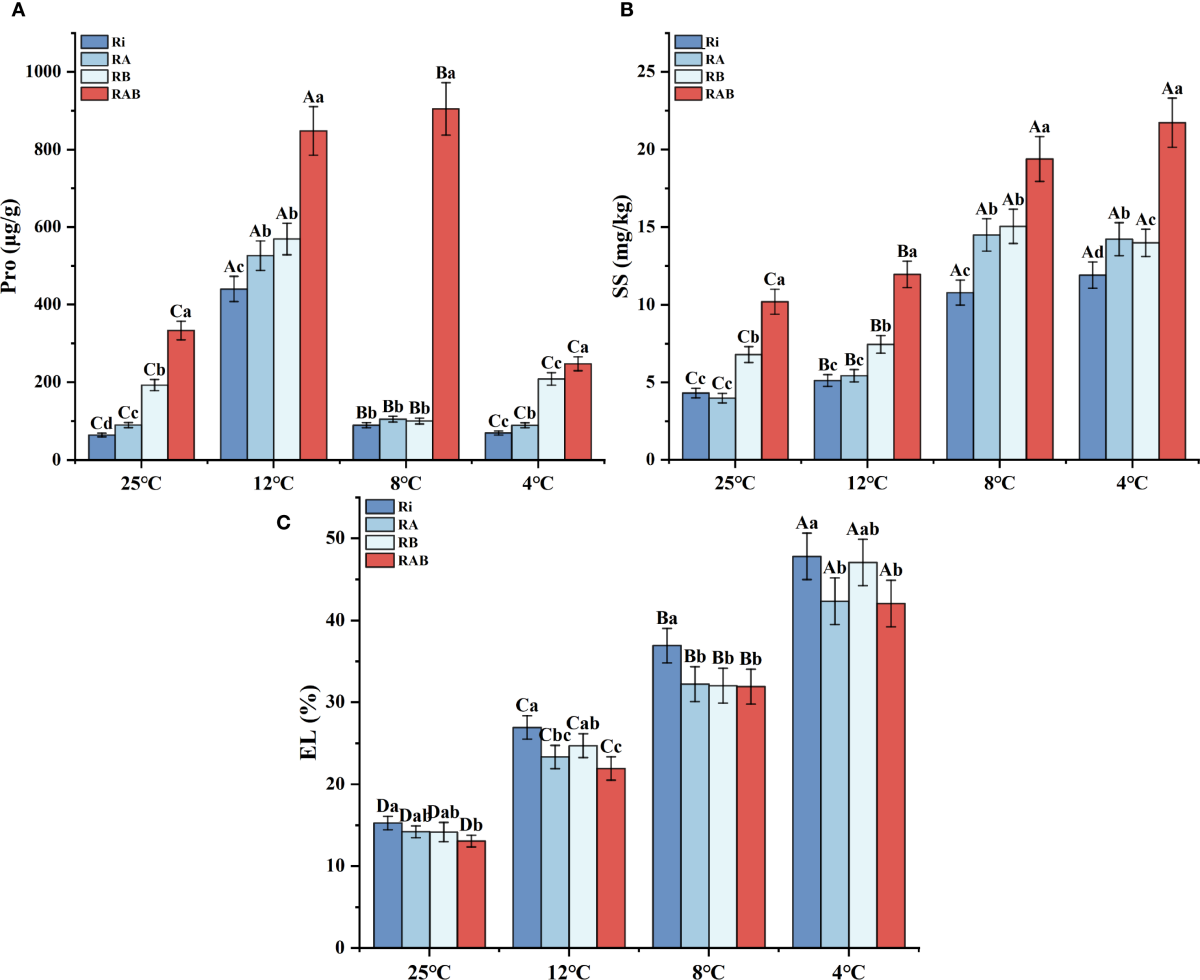
Antioxidant enzyme activity and MDA content in rice seedling leaves. **(A)** Pro; **(B)** SS; **(C)** EL. Results are means ± SD of five replicates. Capital letters represent differences between different temperatures (*P* < 0.05), and lowercase letters represent differences between different fungal agent treatment groups at the same temperature (*P* < 0.05).

Electrolyte leakage (EL) analysis revealed that as the temperature decreased from 25 °C to 4 °C, the EL value in the Ri group increased by 2.13 times, whereas the composite treatments significantly inhibited this increase (*P* < 0.05). Notably, the RAB group exhibited a 13.64% reduction in EL at 4 °C compared to the Ri group, maintaining the most optimal alleviation effect across all temperature gradients (**Figure 3C**). The RA group also demonstrated a significant protective effect (*P* < 0.05).

Dynamic analysis of osmoregulatory substances indicated that proline accumulation exhibited a temperature-dependent biphasic pattern (**Figure 3**). At 12 °C, proline content reached its peak in all treatment groups, with the RAB group showing a 78.4% increase compared to the Ri group (*P* < 0.05). Notably, the accumulation peak for the RAB group occurred at 8 °C, maintaining a 9.9% increase over the peak observed at 12 °C, demonstrating a distinct temperature response pattern. The soluble sugar content increased stepwise with decreasing temperature (**Figure 3B**), stabilizing after 8 °C. The RAB group showed significantly higher accumulation at all temperatures, with the content at 4 °C being 1.82 times that of the Ri group (*P* < 0.05). Furthermore, comprehensive analysis revealed that the osmoregulatory capacity of the RA group was superior to that of the RB group.

### 3.4 Effects of different strain combinations on light gas exchange parameters of rice under cold stress

**Figure 4:**
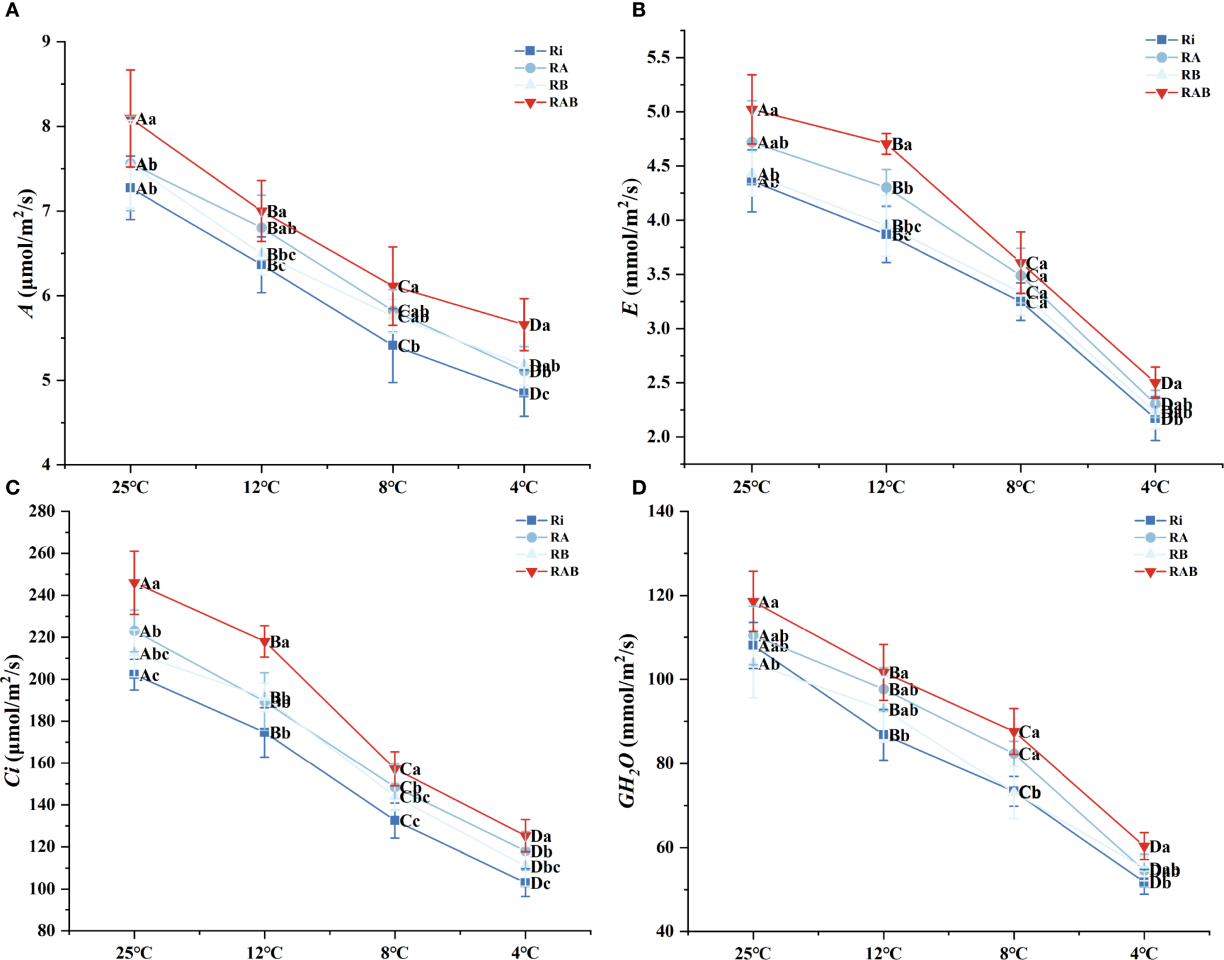
Photosynthetic gas exchange parameters of rice seedling leaves. **(A)** *A*; **(B)** *E*; **(C)** *Ci*; **(D)** *GH_2_O*. Results are means ± SD of five replicates. Capital letters represent differences between different temperatures (*P* < 0.05), and lowercase letters represent differences between different fungal agent treatment groups at the same temperature (*P* < 0.05).

The analysis of photosynthetic physiological characteristics indicates that the net photosynthetic rate (*A*) decreases progressively with a reduction in stress temperature, whereas treatment with the microbial agent significantly mitigates this trend (*P* < 0.05). Specifically, the RAB group exhibited an 11.29% increase in A at 4 °C compared to the Ri group and maintained the highest level across all four temperature gradients. This value was 7.81–9.26% higher than that of the suboptimal treatment group (RB group) (*P* < 0.05) (**Figure 4A**).

The dynamic changes in gas exchange parameters (**Figure 4**) reveal that transpiration rate (*E*), stomatal conductance (*GH_2_O*), and intercellular CO_2_ concentration (*Ci*) exhibit a significant positive correlation with temperature (*P* < 0.05). Under extreme cold stress at 4 °C, the *E*, *GH_2_O*, and *Ci* values in the Ri group decreased by 74.5%, 81.2%, and 63.7%, respectively, compared to those at 25 °C. However, treatment with the microbial agent significantly improved stomatal regulation. Specifically, the RAB group at 4 °C showed increases in *E, GH_2_O*, and *Ci* by 44.3%, 52.6%, and 38.9%, respectively, compared to the Ri group (*P* < 0.05). Notably, the RB group exhibited better performance in terms of net photosynthetic rate and Ci than the RA group.

### 3.5 Effects of different strain combinations on the phytohormone content of rice under cold stress

The content of IAA significantly decreased with decreasing temperature (*P* < 0.05). The addition of the microbial agent significantly increased the IAA levels in rice plants across all temperature conditions (*P* < 0.05). Specifically, the IAA content in the RAB group was significantly higher than that in the Ri group (*P* < 0.05), while no significant difference in IAA content was observed between the RA and RB groups. These findings suggest that the combined application of the microbial agent contributes to the enhancement of plant hormone levels in rice under cold stress.

The levels of ABA (Abscisic acid), GA (Gibberellin), and CTK (Cytokinin) all exhibited a gradual decline with decreasing temperature. Notably, under cold stress conditions, the concentrations of these hormones showed a distinct inhibitory trend. The addition of the microbial agent significantly enhanced the levels of these hormones in rice plants across all temperature conditions. Specifically, the contents of SLS, ABA, GA, and CTK in the RAB group were significantly higher than those in the RA and Ri groups at all temperatures (*P* < 0.05), indicating that the microbial agent effectively regulates the hormonal balance in rice plants. A comprehensive analysis further revealed that the RB group exhibited better promotion of plant hormones than the RA group, with the highest hormone levels observed in the RAB group.

### 3.6 Effects of different strain combinations and cold stress on the relative expression of genes in rice leaves

**Figure 5:**
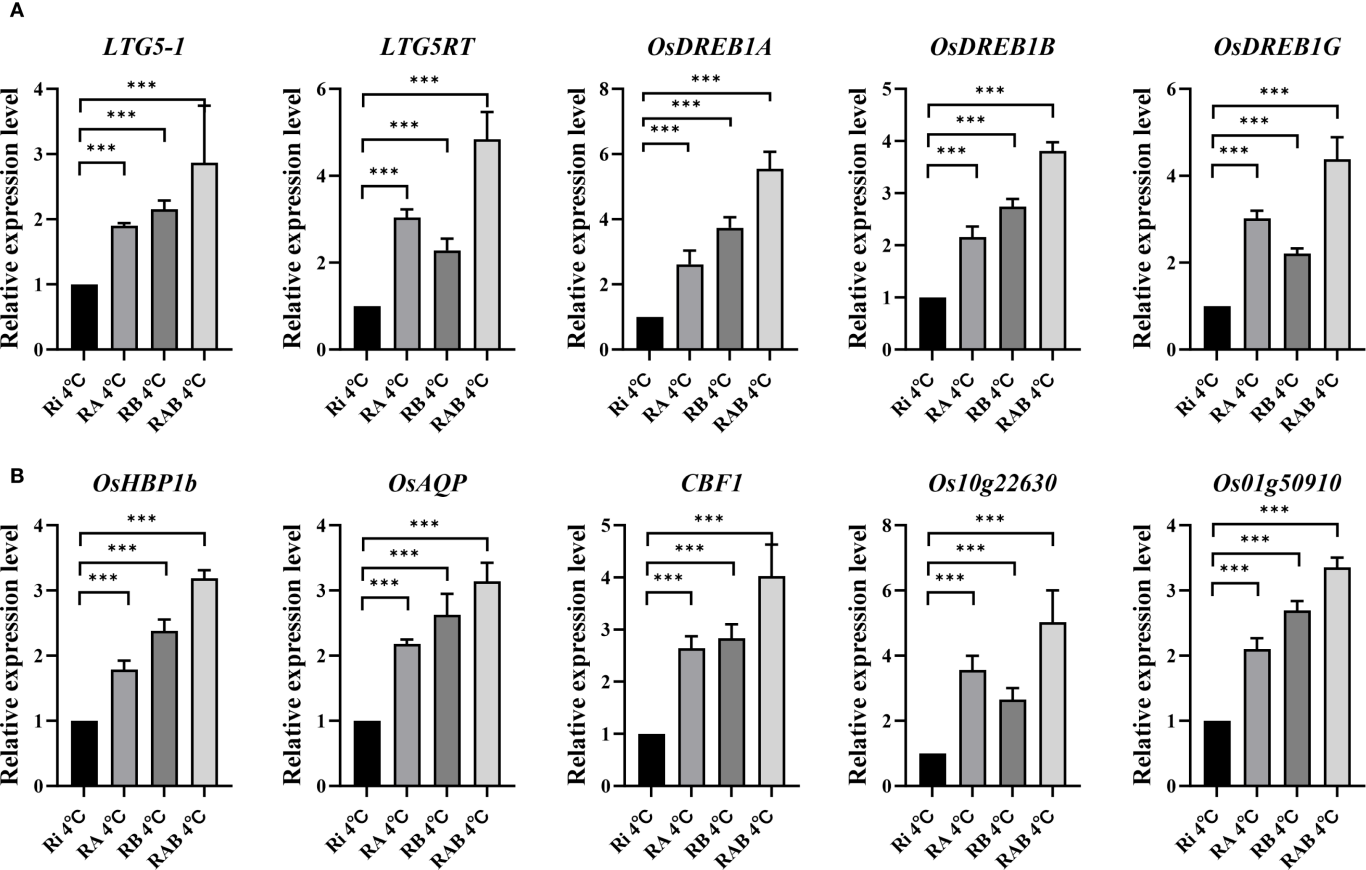
Expression of cold tolerance genes in rice leaves. **(A)** Relative expression of genes related to photosynthesis; **(B)** Relative expression of genes related to cold tolerance; * indicates the difference between different inoculation treatments when the same gene is treated (*P< 0.05; **P< 0.01; ***P< 0.001). ns, not significant.

In this study, qRT-PCR analysis was performed on all treatment groups at 4 °C. Five genes associated with rice cold tolerance were selected for analysis: *LTG5RT, LTG5, OsDREB1A, OsDREB1B*, and *OsDREB1G* (**Figure 5A**). The results of the qRT-PCR analysis revealed that the relative expression levels of the *LTG5RT, LTG5, OsDREB1A, OsDREB1B*, and *OsDREB1G* genes in the RA, RB, and RAB groups were significantly higher than those in the Ri group (*P* < 0.001). Additionally, it was found that the expression levels of *LTG5RT* and *OsDREB1G* in the RA group were higher than those in the RB group, indicating that the combined application of Ar and AMF has a more pronounced effect on enhancing rice cold tolerance compared to Bs.

**Figure 6:**
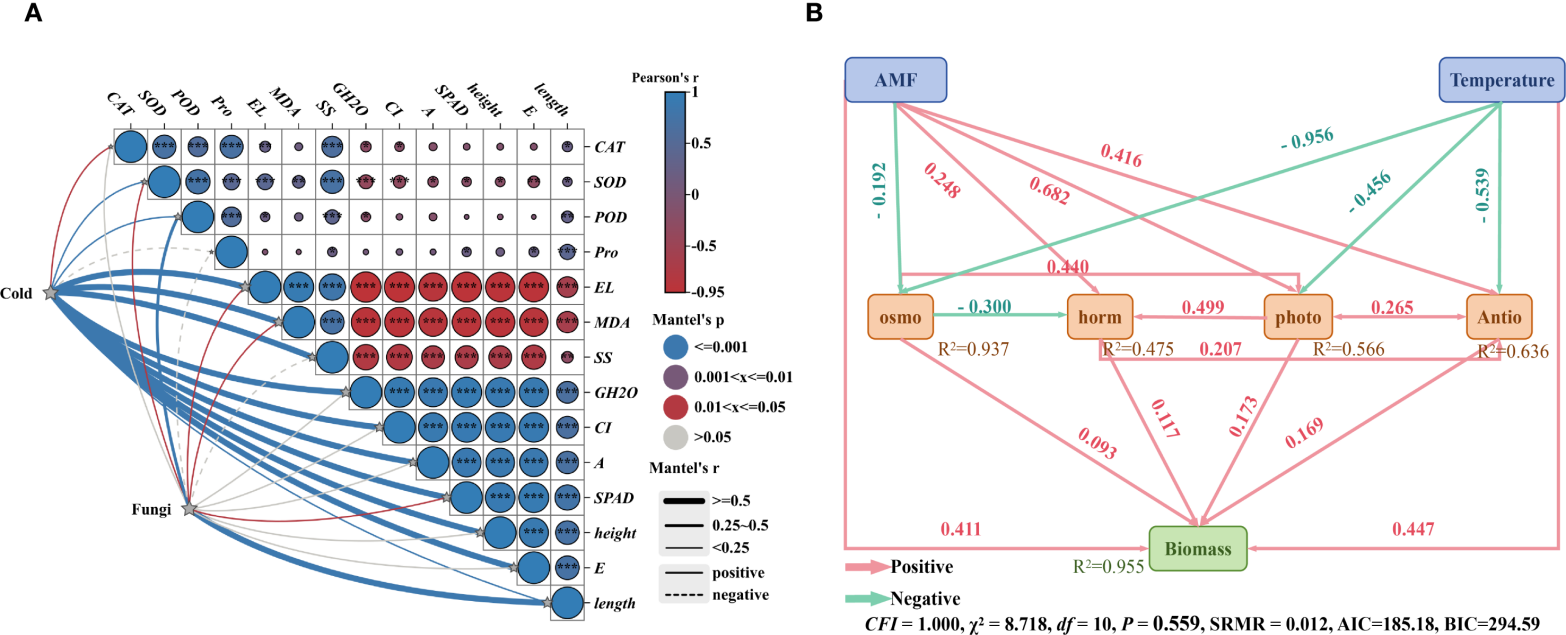
Structural equation model diagram of the effects of fungal agent addition and temperature on rice plant height, root length, and net photosynthetic rate. **(A)** Mantel test diagram; **(B)** Structural equation model diagram. AMF: Ri, RA, RB, RAB; Temperature: 25 °C, 12 °C, 8 °C, 4 °C; osmo: Osmoregulation; horm: Phytohormones; photo: Photosynthesis; Antio: Antioxidant enzymes. The numbers along the arrows represent path coefficients, with pink lines indicating positive path coefficients and green lines indicating negative path coefficients.

Five genes related to the photosynthetic gas exchange parameters of rice under cold stress were selected for analysis (**Figure 6B**): *OsHBP1b, OsAQP, CBF1, Os10g22630*, and *Os01g50910*. The results of the qRT-PCR analysis showed that the relative expression levels of *OsHBP1b, OsAQP, CBF1, Os10g22630*, and *Os01g50910* in the RAB treatment group were significantly different from those in the Ri group (*P* < 0.01). Furthermore, it was observed that the expression levels of *OsAQP* and *OsHBP1b* in the RB group were higher than those in the RA group.

### 3.7 Relationship between rice seedling biomass, fungal agent addition, and cold stress

Physiological and biochemical analyses were conducted to investigate the direct and indirect effects of microbial agent application and cold stress on rice seedling biomass (**Figure 6**). A Mantel test was performed on these parameters, revealing that both cold treatment and microbial agent application significantly influenced these parameters (*P* < 0.05). A structural equation model was constructed based on the results, and the final model met our significance criteria (*CFI* = 1.000, χ^2^ = 8.718, *df* = 10, *P* = 0.559, *SRMR* = 0.012). The addition of the microbial agent and cold stress together accounted for 95.5% of the variation in biomass. The microbial agent, temperature, antioxidant defense, osmoregulation, photosynthesis, and phytohormones had significant direct and indirect effects on biomass.

## 4 Discussion

### 4.1 Effects of different strain combinations on AMF colonization and biomass of rice under cold stress

In this study, the mycorrhizal colonization rate of the RA group, RB group, and RAB group with mycorrhizal helper bacteria added was higher than that of the Ri group. This is consistent with the results of Ji who found that inoculation with *Bacillus subtilis* under low salt stress can further promote AMF colonization and increase wheat yield (Ji et al., 2022). This further proves the helper effect of the two mycorrhizal helper bacteria on AMF, and there is also a significant increase in plant biomass, achieving better results.

The rice seedling stage is the most sensitive to cold stress, so studying the cold tolerance of rice seedlings is an important indicator for exploring the cold tolerance of rice throughout the growth period (Pan et al., 2023). Biomass is an important indicator for evaluating the cold tolerance of rice seedlings. Under low-temperature conditions, the growth rate of rice seedlings is significantly slowed down (Guo et al., 2022). Cold stress can inhibit cell division and elongation, limiting the development of roots, stems, and leaves, resulting in a reduction in biomass (Song et al., 2021; Wang et al., 2023a). In this study, cold stress significantly reduced the plant height and root length of rice seedlings compared to normal temperature treatment, and the addition of inoculants alleviated this damage (**Figure 2**). At each treatment temperature, the addition of inoculants increased the plant height and root length of rice seedlings. Even under mild cold stress, the plant height and root length of rice seedlings in the RAB treatment group exceeded that of the normal temperature treatment, which showed that the RAB treatment group greatly improved the cold tolerance of rice seedlings. This is consistent with previous research results (Liu et al., 2022a). It may be that compared with inoculating Ri alone and not inoculating bacteria, inoculating Ri and two mycorrhizal helper bacteria further increased the root area and nutrient absorption capacity of rice seedlings. And it reduces the damage to rice seedling cells caused by cold stress.

The three microorganisms in this study play a synergistic role in improving the cold tolerance of rice. Compound inoculation improved the cold tolerance of rice seedlings better than adding Ri alone. The hairy roots formed by Ar-induced plants can coexist with the mycorrhizal structure of AMF in the plant root system (Ramasamy et al., 2023; Wang et al., 2023b). Studies have shown that the mycorrhizal structure of AMF can promote the formation and growth of Ar-induced hairy roots and increase the number and branching degree of hairy roots (Landi et al., 2009). Moreover, the induction of Ar can promote the colonization of host plants by AMF, and enhance the stress tolerance of plants by regulating the physiological processes of plants (Pandit et al., 2022). *Bacillus subtilis* is a common plant growth-promoting bacterium that can enhance plant stress resistance by inducing plant defense systems (Yadav et al., 2021). Bs can produce some substances, such as hormones and growth factors. These substances can promote AMF to function better (Hashem et al., 2016). AMF may induce plant roots to secrete some substances, providing nutrients and a more suitable ecological environment for Bs (Zhou et al., 2022a). Collectively, our findings provide a robust theoretical foundation for the development of multi-strain microbial consortia as a sustainable agricultural technology. The significant improvement in biomass and colonization rates under cold stress demonstrates the tangible benefits of this synergistic relationship.

### 4.2 Effects of different strain combinations on MDA content and antioxidant enzyme activities of rice under cold stress

In this study, cold stress increased the MDA content in rice seedlings, indicating that cold stress caused oxidative damage to rice cells, and the degree of damage continued to increase as the temperature decreased (**Figure 3D**). Cold stress can cause plants to produce and accumulate more reactive oxides, such as superoxide anions, hydrogen peroxide, and hydroxyl radicals (Liu et al., 2023b). These reactive oxides can cause oxidative damage to biological molecules such as cell membranes, proteins, and nucleic acids at high concentrations (Qinhan et al., 2022). The addition of strains in the treatment groups of this experiment will reduce this damage, and the MDA content in each treatment group is lower than that in the Ri group. This is consistent with the research results of Liu et al (Liu et al., 2016). The MDA content in the RAB treatment group was the lowest, which shows that the addition of two mycorrhizal helper bacteria and Ri can improve the ability of rice seedlings to withstand cold stress better than adding Ri alone. It further clarified the important role of research on compound fungi in improving rice stress resistance.

In this study, rice seedlings produced more antioxidant enzymes to reduce oxidative damage caused by cold stress. As the temperature decreases, the SOD, POD, and CAT activities of rice seedlings increase (**Figure 3**). Moreover, the antioxidant enzyme activity of rice seedlings in the fungal agent-added group was higher than that in the Ri group, with the RAB group having the highest antioxidant enzyme activity. This is similar to the results obtained by Chu (Chu et al., 2016). In plants, SOD is mainly responsible for converting superoxide free radicals into more stable hydroperoxides (Roshan et al., 2022), while POD is responsible for converting peroxides into water, and CAT is accountable for converting highly oxidizing hydrogen peroxide into water and oxygen (Yang et al., 2023a). These three enzymes interact to form an antioxidant defense line that works together within cells to protect cells from oxidative damage (Tavanti et al., 2021). The results of this study show that the addition of Ri agents can enhance the antioxidant defense system of rice seedlings under cold stress and reduce the damage caused by cold stress. And Ri works best when used together with two mycorrhizal helper bacteria.

### 4.3 Effects of different strain combinations on EL and osmotic regulatory substance content in rice under cold stress

Cold stress can cause the crystallization of plant cell membranes and cause intracellular electrolyte leakage (Lainé et al., 2023). In this study, as the treatment temperature decreased, the EL of rice leaves in each group gradually increased. This shows that cold stress has caused varying degrees of damage to rice seedlings. The lower the temperature, the greater the degree of damage (**Figure 4C**). However, the EL of rice leaves in the RI group, RA group, RB group, and RAB group were lower than that of the Ri group at different temperatures, and the RAB group had the lowest EL. This is consistent with the research results of Balasjin et al., who found that cold stress can increase the EL of rice, while adding plant growth promoting bacteria can reduce the EL of rice (Balasjin et al., 2023). This shows that the addition of Ri agents will reduce electrolyte leakage caused by cold stress and protect the integrity of the cell membrane of rice seedlings. This may be because AMF can expand the absorption range of plant roots, absorb and transport nutrients such as water and electrolytes from the soil through its hyphal network, and maintain the osmotic balance inside and outside the cell (Bennett and Groten, 2022). Moreover, the symbiosis between AMF and plant roots will produce a series of reactions, including thickening of cell walls, synthesis of antioxidant substances, and scavenging of reactive oxygen species, which help protect the integrity and functional stability of plant cell membranes and reduce electrolyte leakage (Kokkoris et al., 2020). In addition, the effect of co-adding Ri and mycorrhizal helper bacteria is better than using Ri alone, so in future research, priority should be paid to the study of composite inoculants.

In this study, the intracellular Pro and SS contents of rice seedlings changed differently as the temperature decreased (**Figures 4A, B**). The rice Pro and SS contents in the Ri group, RA group, RB group, and RAB group were significantly higher than those in the Ri group. Especially in the RAB group, the Pro content reaches the highest at 8 °C, which is different from other groups where the Pro content reaches the highest at 12 °C and then decreases. It shows that adding two kinds of mycorrhizal helper bacteria and the complex inoculants formed by Ri can significantly improve the cold tolerance of rice seedlings. The research results of Zhang found that inoculation with AMF compound inoculants under salt stress can increase the Pro content of rice seedlings under salt stress, but there are limitations in the ability to increase (Zhang et al., 2023a). This study also achieved similar results. This may be because, under stress, plants will reduce the supply of sugars to AMF, thereby affecting the number and function of mycorrhizal fungi and limiting their ability to enhance plant stress resistance (Wipf et al., 2019). Although AMF can enhance plant stress resistance through some mechanisms, these mechanisms may only be effective for specific stress types or specific plant species and have limited effects on other stress conditions or plant species (Wu et al., 2012). Therefore, our results advocate for a shift in research and development towards formulating tailored microbial consortia, rather than relying on single strains. The proven efficacy of the RAB combination makes it a prime candidate for commercialization as a bio-stimulant to enhance cold tolerance in rice seedlings. This strategy holds significant promise for improving crop establishment and yield stability in temperate and high-altitude rice-growing regions prone to unseasonal cold snaps.

### 4.4 Effects of different strain combinations on photosynthetic gas exchange parameters of rice under cold stress

Cold stress primarily reduces the photosynthetic capacity of plants by disrupting photosynthetic organs and decreasing the content of photosynthetic pigments (Xu et al., 2020). Chloroplasts, as the key sites for photosynthesis, are critical for this process, and the chlorophyll content is one of the main indicators of a plant’s photosynthetic ability (Chang et al., 2021). This study found that cold stress reduced the *A* of rice seedlings, while inoculation with Ri enhanced photosynthetic activity in the seedlings, resulting in an increase in *A*. And the effect of adding different strains in combination was higher than that of using them alone. It indicates that the compound addition of mycorrhizal helper bacteria and Ri can reduce the destruction of chloroplasts of rice seedling cells by cold stress, protect the integrity of rice seedling cells, and improve their photosynthesis and cold tolerance.

**Figure 7:**
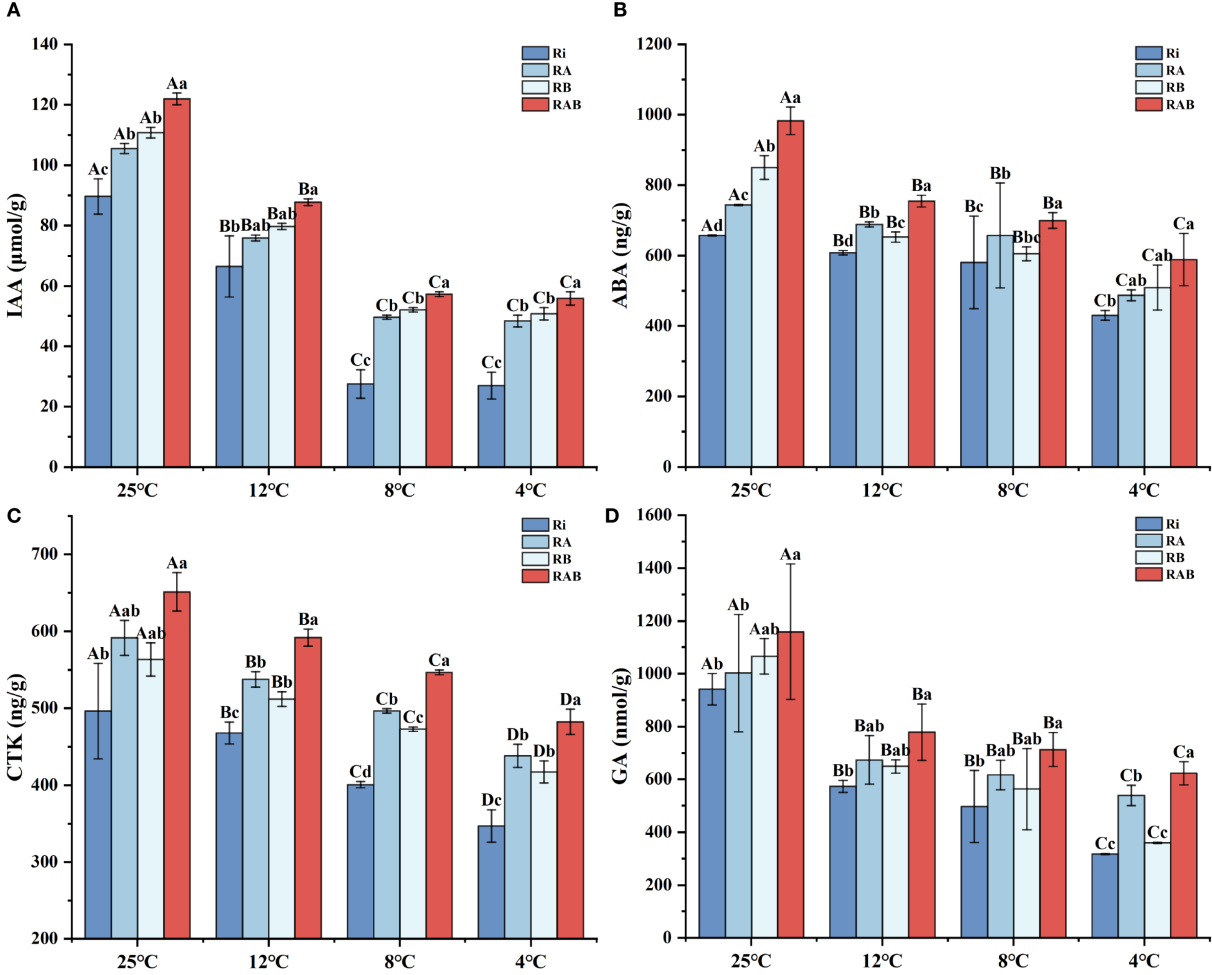
Phytohormone content of rice seedling leaves. **(A)** IAA content; **(B)** ABA content; **(C)** CTK content; **(D)** GA content. Results are means ± SD of five replicates. Capital letters represent differences between different temperatures (*P* < 0.05), and lowercase letters represent differences between different fungal agent treatment groups at the same temperature (*P* < 0.05).

Photosynthesis is an important physiological process that affects plant growth. Studies have shown that inoculation with AMF can increase the photosynthesis intensity of plants (Frosi et al., 2018). The research results of Liu et al. found that those inoculated with AMF showed a higher net photosynthetic rate under stress conditions, and the degree of chloroplast damage was less (Liu et al., 2023c). This experiment also achieved similar results. The results of this experiment found that under cold stress conditions, the *A, E, Ci, and GH_2_O* of rice leaves in the Ri group, RA group, RB group, and RAB group were all higher than those in the Ri group (**Figure 7**). Mycorrhizalizal rice may improve photosynthetic capacity by increasing photosynthetic gas exchange capacity and water absorption, thereby mitigating the damage caused by cold stress. This shows that inoculation of mycorrhizal helper bacteria under cold stress further helps Ri improve the water use efficiency and light energy efficiency of rice, and further improves the cold tolerance of rice seedlings.

### 4.5 Effects of different strain combinations and cold stress temperatures on phytohormone levels in rice

The plant hormone network plays a central regulatory role in crop responses to environmental stress (Liu et al., 2023a). This study found that cold stress significantly inhibited the synthesis of IAA, ABA, GA, and CTK in rice, which is consistent with previous reports on the suppression of hormone biosynthesis under cold stress (Yang, 2022). The IAA content exhibited a gradient decline with decreasing temperature, which aligns with key findings regarding the suppression of the tryptophan aminotransferase (TAA1/YUC) pathway under cold stress (Kalamulla et al., 2022). Notably, microbial agent treatment (especially the RAB group) reversed this trend, likely due to the induction of indole-3-pyruvic acid (IPA) precursor synthesis by Ri (Zhou et al., 2022b). Additionally, the ACC deaminase produced by *Bacillus subtilis* reduced ethylene levels (Kono et al., 2022), thereby alleviating the ethylene-induced inhibition of IAA synthesis. This synergistic effect resulted in a significant increase in IAA content in the RAB group at 4 °C compared to the Ri group, effectively maintaining the auxin levels required for root morphology development and leaf expansion.

Cold stress is one of the important environmental factors affecting the growth and development of rice seedlings, significantly interfering with their phytohormone synthesis and signaling (Jarosová et al., 2024). Under cold stress, the synthesis of ABA in rice seedlings was significantly reduced, resulting in inhibited root growth and suppression of the plant’s vertical growth (Cai et al., 2023). Under cold stress, the synthesis pathway of GA was inhibited, reducing the cell elongation capacity, while the decreased synthesis of CTK affected cell division and differentiation (Tang et al., 2022). This resulted in stunted plant growth and yellowing of leaves. Both of these hormones play crucial roles in the response to biotic and abiotic stresses (Liu et al., 2022b). This study found that cold stress reduced the levels of plant hormones in rice seedlings, while the addition of microbial agents mitigated this damage, with the RAB group showing significantly higher levels than the other groups. This may be attributed to the ability of mycorrhizal symbionts to synthesize and secrete precursor substances of certain plant hormones (Noctor and Foyer, 2016). These substances can be absorbed and utilized by the host plant, directly increasing the levels of hormones within the plant (Akhoundnejad and Baran, 2023).

### 4.6 Effects of different strain combinations on the relative expression of cold-tolerance genes in rice under cold stress

*CBF* genes can promote the cold tolerance of plants by activating the expression of cold resistance-related genes (Kopec et al., 2022). Cold tolerance is necessary for plants to carry out normal photosynthesis in cold environments (Yamori et al., 2010). Therefore, the *CBF* gene can ensure the progress of photosynthesis by enhancing the cold tolerance of plants. In addition, *CBF* genes are also involved in regulating physiological processes such as water utilization and nutrient metabolism in plants, further affecting the efficiency and stability of photosynthesis (Shi et al., 2018). Therefore, *CBF* genes have important functions in the regulation of plant photosynthesis. In this study, a qRT-PCR experiment was conducted on the *CBF1* gene. And it was found that RAB treatment significantly increased the relative expression of the *CBF1* gene in rice seedlings at 4 °C, and also up-regulated the expression of other photosynthesis-related genes (**Figure 5A**). Wang et al. found that the photochemical efficiency (*Fv/Fm*) and photooxidation efficiency of transgenic plants overexpressing *CBF1* under low-temperature stress were higher than those of wild-type plants (Yang et al., 2010), which is consistent with the results of this study. This verifies that the combined addition of mycorrhizal helper bacteria and Ri can make rice seedlings have higher photosynthetic intensity and SPAD value under cold stress.

*LTG5* and *LTG5RT* genes are cold-tolerant genes encoding UDP-glucosyltransferase. Plants need UDP-glucosyltransferase to glycosylate plant hormones and other plant secondary metabolites. This gene plays a role in plant resistance to abiotic stress, regulating the effect (Dong and Hwang, 2014; Neilson et al., 2017). Relevant studies have shown that overexpression of the *LTG5* gene improves the germination rate of rice during the germination period and enhances the cold tolerance of rice (Pan et al., 2020). Moon found that the DREB subfamily plays an important role in plant cold tolerance, among which *OsDREB1G* is specifically induced under cold stress conditions (Moon et al., 2019). *OsDREB1G* is a typical *CBF/DREB1* transcription factor with specific functions in cold stress response. In this study, qRT-PCR experiments were conducted on genes such as *LTG5, LTG5RT, OsDREB1A, OsDREB1B*, and *OsDREB1G*. It was found that the relative expression of cold-tolerance genes in rice seedlings at 4 °C in the RAB treatment group was significantly increased (**Figure 5B**). It was verified that mycorrhizal rice has stronger cold tolerance under cold stress. This study shows that the co-addition of helper bacteria and Ri plays an important role in improving the cold tolerance of rice seedlings, which provides a theoretical basis for further revealing the regulatory mechanism of the combination of helper bacteria and mycorrhizal fungi on rice growth under cold stress.

## 5 Conclusion

This study elucidates the synergistic mechanism by which mycorrhizal-helper bacteria (MHB) and arbuscular mycorrhizal fungi (AMF) collaboratively enhance the cold tolerance of rice. Co-inoculation with Ri, Bs, and Ar significantly enhanced the physiological homeostasis of rice seedlings under cold stress by stimulating antioxidant defense, optimizing osmotic regulation, and maintaining hormonal balance. The synergistic effect manifested as increased biomass, improved photosynthetic efficiency, and differential activation of cold tolerance genes (*CBF1, LTG5RT*, and *OsDREB1B*). Functional partitioning analysis revealed that Bs primarily promotes photosynthetic carbon assimilation and biomass accumulation, while Ar more significantly enhances antioxidant metabolism and the expression of cold tolerance genes. This finding provides new insights into the principles underlying microbial synergistic effects. Additionally, it offers theoretical and technical support for ensuring stable rice yields in cold regions and safeguarding food security under climate change, holding significant ecological agricultural application value.

## Data Availability

The original contributions presented in the study are included in the article/**Supplementary Material**. Further inquiries can be directed to the corresponding author.
