# Supplementary material for: Mycorrhizal helper bacteria further promote mycorrhizal fungi to improve cold tolerance in rice seedlings: evidence from oxidative stress, osmoregulation, photosynthesis, and related genes in rice
Source: Front Plant Sci. 2025 Oct 15;16:1692304. doi: 10.3389/fpls.2025.1692304 (PMC12572616; doi:10.3389/fpls.2025.1692304)
Supplement: Supplementary file 1 [file Table1.docx]

**Supplemental Table S1. Primer Sequence and Annealing Temperature**

| Gene name | Primer sequence | Annealing  Temp. (◦C) | References |
| --- | --- | --- | --- |
| *OsDREB1G* | For（5′- CCACTAATTCGAACGCCGAAC-3′） | 56.7 | (Pan et al. 2020)  (Moon et al. 2019) |
|  | Rev（5′- GCTACCTACGCAGGATCAC-3′） | 55.5 |  |
| *Os10g22630* | For（5′- CCCATCGTCGGTAGTCACTG-3′） | 58.0 |  |
|  | Rev（5′- AGGAGCCACAGGAGATGCTA-3′） | 58.1 |  |
| *OsDREB1A* | For（5′- GACGTCCTGAGTGACATGGG-3′） | 58.3 |  |
|  | Rev（5′- AGTAGCTCCAGAGTGGGACG-3′） | 59.1 |  |
| *OsDREB1B* | For（5′- CTCGCACTGAAAAGTGTGGAC -3′） | 56.6 |  |
|  | Rev（5′- GGAGGGAGAAATCTGGCACA -3′） | 57.3 |  |
| *Os01g50910* | For（5′- AGGGGAGCAGGTGAAGAG -3′） | 57.0 |  |
|  | Rev（5′- TGTAGGTGCTGGTGTCCTT -3′） | 56.0 |  |
| *OsHBP1b* | For（5′- CGACATCGAGGTCAAGCTCA -3′） | 57.3 |  |
|  | Rev（5′- CAGACTTTGCAGTCGGTGC-3′） | 57.2 |  |
| *LTG5-1* | For（5′- ATGACGACAAAGACCTTT-3′） | 47.6 |  |
|  | Rev（5′- TCAGCTGCGAACTCCATT-3′） | 54.0 |  |
| *OsAQP* | For（5′- GCCTATTTCTACGCCAACG-3′） | 53.8 |  |
|  | Rev（5′- CCACTTGAGCACCCACAGC-3′） | 60.0 |  |
| *LTG5RT* | For（5′- CCCCGCCTACTTCTTCTTTC-3′） | 55.7 |  |
|  | Rev（5′- CGCCGCCTTATCCATCTC-3′） | 56.3 |  |
| *CBF1* | F（5′- AAGGACCAAGTTCAGGGAGACGAGG-3′） | 62.9 |  |
|  | R（5′- CGGAGTCGGCGAAGTTGAGG-3′） | 61.6 |  |

**Reference**

Moon SJ, Min MK, Kim JA, Kim DY, Yoon IS, Kwon TR, Byun MO, Kim BG (2019) Ectopic Expression of OsDREB1G, a Member of the OsDREB1 Subfamily, Confers Cold Stress Tolerance in Rice. Front Plant Sci 10. doi:10.3389/fpls.2019.00297

Pan YH, Liang HF, Gao LJ, Dai GX, Chen WW, Yang XH, Qing DJ, Gao J, Wu H, Huang J, Zhou WY, Huang CC, Liang YT, Deng GF (2020) Transcriptomic profiling of germinating seeds under cold stress and characterization of the cold-tolerant gene LTG5 in rice. Bmc Plant Biol 20 (1). doi:10.1186/s12870-020-02569-z
