# Supplementary material for: Mycorrhizal helper bacteria further promote mycorrhizal fungi to improve cold tolerance in rice seedlings: evidence from oxidative stress, osmoregulation, photosynthesis, and related genes in rice
Source: Front Plant Sci. 2025 Oct 15;16:1692304. doi: 10.3389/fpls.2025.1692304 (PMC12572616; doi:10.3389/fpls.2025.1692304)
Supplement: Supplementary file 2 [file Table2.docx]

**Supplemental Table S2. qRT-PCR reaction conditions**

| Stage1 | pre-denatured | Reps：1 | 95℃ | 30sec |
| --- | --- | --- | --- | --- |
| Stage2 | cyclic reaction | Reps：40 | 95℃ | 10sec |
|  |  |  | 60℃ | 30sec |
|  |  |  | 95℃ | 15sec |
| Stage3 | Melting curve | Reps：1 | 60℃ | 60sec |
|  |  |  | 95℃ | 15sec |
